# CYP3A5 and UGT1A9 Polymorphisms Influence Immunosuppressive Therapy in Pediatric Kidney Transplant Recipients

**DOI:** 10.3389/fphar.2021.653525

**Published:** 2021-04-22

**Authors:** Paola Krall, Dominique Yañez, Angélica Rojo, Ángela Delucchi, Miguel Córdova, Jorge Morales, Pía Boza, Alonso de la Rivera, Natalie Espinoza, Natalia Armijo, Luis E. Castañeda, Mauricio J. Farfán, Carolina Salas

**Affiliations:** ^1^ Departamento de Pediatría y Cirugía Infantil Oriente, Facultad de Medicina, Universidad de Chile, Santiago de Chile, Chile; ^2^ Laboratorio Clínico, Hospital Luis Calvo Mackenna, Santiago de Chile, Chile; ^3^ Servicio de Farmacia, Hospital Luis Calvo Mackenna, Santiago de Chile, Chile; ^4^ Unidad de Nefrología, Hospital Luis Calvo Mackenna, Santiago de Chile, Chile; ^5^ Programa de Genética Humana, Instituto de Ciencias Biomédicas, Facultad de Medicina, Universidad de Chile, Santiago de Chile, Chile

**Keywords:** pediatric kidney transplantation, pharmacogenetics, tacrolimus, mycophenolic acid, pharmacokinetics

## Abstract

**Background:** Tacrolimus (TAC) and mycophenolic acid (MPA) are the main immunosuppressive drugs used in pediatric kidney transplantation. Single nucleotide polymorphisms (SNPs) in metabolizing enzymes and transporters might influence plasma levels of these drugs. Herein, we sought to determine the influence of SNPs on *CYP3A5*, *MRP2* and *UGT1A9* genes in Chilean pediatric kidney recipients using TAC and MPA.

**Patients and Methods:** A prospective study was performed on 104 pediatric kidney recipients that used TAC and MPA for immunosuppression. The median age at the time of transplantation was 8.1 years [Q1–Q3 4.5–11.6 years] and the main clinical diagnosis was a structural anomaly. In a subgroup of patients, a complete steroid withdrawal was made at day 7. The *CYP3A5* polymorphism (ancestral allele *1; variant allele *3) was determined in the entire cohort, while *MRP2* -24G > A, *UGT1A9* -275T > A, and *UGT1A9* -2152C > T polymorphisms were determined in 53 patients. Genotypes were associated with trough drug concentrations (C_0_), dose requirements normalized by weight (TAC-D mg/kg) or body surface (MPA-D mg/m2), trough levels normalized by dose requirements (C_0_/D), and area under the curve in 12 h normalized by dose requirements (AUC_0–12h_/D).

**Results:** The frequencies of the variant alleles CYP3A5*3, *MRP2-24A*, *UGT1A9-275A*, and *UGT1A9-2152T* were 76.9, 22.1, 6.6, and 2.9%, respectively. AUC_0–12h_/TAC-D were 1.6-fold higher in CYP3A5*3/*3 patients than in CYP3A5*1 carriers (CYP3A5*1/*3 and CYP3A5*1/*1). When analyzing patients with steroid withdrawal, CYP3A5*3/*3 patients had 1.7-fold higher AUC_0–12h_/TAC-D than the other genotypes. Patients carrying the CYP3A5*3/*3 genotype had higher TAC-C_0_, lower TAC-D and higher TAC-C_0_/D, consistently in a 6-months follow-up. Creatinine clearance was stable during the follow-up, regardless of the genotype. No significant differences between *MRP2* and *UGT1A9* genotypes were observed in MPA-C_0_, MPA-D or MPA-C_0_/D. However, patients carrying the *UGT1A9-275A* allele had lower AUC_0–12h_/MPA-D than those carrying the *UGT1A9-275T* ancestral allele.

**Conclusions:** These results support that *CYP3A5* and *UGT1A9* genotyping in pediatric recipients might be useful and advisable to guide TAC and MPA dosing and monitoring in children that undergo kidney transplantation.

## Introduction

Kidney transplantation is the renal replacement therapy of choice in the pediatric population, improving survival, growth and quality of life (McDonald and Craig, 2004). Immunosuppressive therapy to avoid rejection is the cornerstone of graft survival (Winterberg and Garro, 2019). Tacrolimus (TAC) and mycophenolic acid (MPA) are the main immunosuppressive drugs used in pediatric kidney transplantation in combination with steroids. However, an early tapering of steroids until complete withdrawal maintaining the TAC and MPA dosage in pediatric recipients with low immunological risk is associated with a better growth pattern (Delucchi et al., 2007).

TAC and MPA need continuous monitoring to verify that the plasma concentrations are within the therapeutic ranges, to maintain the balance between efficacy and toxicity and, consequently, to ensure graft and patient survival (Zwart et al., 2018). The drug bioavailability might be influenced by genetic variations, such as single nucleotide polymorphisms (SNPs) in metabolizing enzymes or transport proteins, causing increases or decreases in plasmatic drug concentrations (Golubovic et al., 2016).

TAC is a calcineurin inhibitor that is metabolized by the cytochrome P450 enzymes, mainly CYP3A4 and CYP3A5, involved in the first step of drug metabolism. CYP3A5 plays the most important role when a dose of this drug is prescribed, particularly in pediatric patients (Zheng et al., 2003; De Wildt et al., 2011). Several studies have shown that the CYP3A5 expression is correlated with reduced TAC plasma levels and a delay in achieving adequate plasma concentrations with the conventional exposure dose, which increases the risk of acute rejection incidence (Kuehl et al., 2001; Staatz, Taylor, and Tett 2001; Macphee et al., 2002; MacPhee et al., 2004; Min et al., 2010). By contrast, recipients treated with TAC who do not express this enzyme are more exposed to nephrotoxicity that might be explained by plasma TAC accumulation (Thervet et al., 2003).

The variability in *CYP3A5* enzyme expression is explained mainly by the *CYP3A5* gene that contains a SNP described as a nucleotide change located in intron 3 (rs776746; *CYP3A5* c. A6986G). This genetic variation results in two alleles: the ancestral allele called CYP3A5*1 and the variant allele called CYP3A5*3. The presence of two CYP3A5*3 alleles (CYP3A5*3/*3) results in a truncated protein as a consequence of alternative splicing causing the absence of enzyme activity, also known as a non-expressor genotype. On the other hand, the presence of at least a single CYP3A5*1 allele (CYP3A5*1/*3 or CYP3A5*1/*1) is related to a functional enzyme, and these genotypes are cataloged as expressors (Kuehl et al., 2001). Based on genetic studies, the frequency of the CYP3A5*3 allele displays variability according to the geographic and ethnic origin, ranging from 32% in African individuals to 93% in European individuals. This variability reinforces the need to analyze the frequency of the *CYP3A5* genotype and the association with plasma TAC levels in different human geographic populations or ethnic groups (Xie et al., 2004).

MPA is an inhibitor of T-cell proliferation strongly influenced by the metabolizing diphosphate glucuronyl transferase (UGT) enzymes and the multidrug resistance-associated proteins (MRP) that act as drug transporters on the membrane of hepatocytes (Barraclough et al., 2010). Similar to *CYP3A5*, there is genetic variability and several reports have demonstrated that the specific variants *MRP2* -24C (rs717620), *UGT1A9* -275A (rs6714486) and *UGT1A9* -2152T (rs17868320) polymorphisms might have a role in the inter-individual variability of MPA plasma levels in pediatric organ recipients (Bernard and Guillemette, 2004; Naesens et al., 2006; Fukuda et al., 2012).

Considering that polymorphisms involved in the metabolism of TAC and MPA have a geographic and ethnic variability, in this study we aimed to determine the frequency of SNPs in *CYP3A5*, *MRP2*, and *UGT1A9* genes in Chilean pediatric kidney recipients. Additionally, we evaluated the influence of these polymorphisms on TAC and MPA plasma levels. Our data support that the *CYP3A5* and *UGT1A9* genotyping in pediatric recipients might be useful and advisable to guide TAC and MPA dosing and monitoring in children that undergo kidney transplantation.

## Patients and Methods

### Patient Data

Pediatric patients that underwent kidney transplantation with a deceased or living-related donor between 2001 and 2019 at the referral transplant center Hospital Luis Calvo Mackenna (Santiago, Chile) were invited to participate in this descriptive, prospective, longitudinal study. In our center, 85–90% of the transplantations were performed with deceased donors each year. We studied 104 recipients who received TAC and MPA as immunosuppressive therapy combined with steroids. Patients were induced with basiliximab on day 0 during surgery before reperfusion and on day 4 post-transplantation. TAC (starting dose 0.15 mg/kg twice per day), MPA (starting dose 800 mg/m2 twice per day), and steroids were administered according to the institutional protocol. Steroid use was individualized according to immunological risk and graft function in each patient. Starting steroid therapy was methylprednisolone 2 mg/kg/day for 2 days and switched to prednisone at day 3. The patients undergoing steroid withdrawal were treated successively with 2, 1, 0.5, and 0.25 mg/kg/day, each dose for one day. The patients that continued steroid therapy switched to prednisone 2 mg/kg/day at day 3 and reduced gradually until reaching 0.12 mg/kg/day at month 6, and this was kept constant from that month onwards. Therapeutic drug monitoring of TAC was applied regularly to all recipients and dose adjustment was performed when necessary to achieve target trough levels (10–15 ng/ml in the first 3 months and 5–7 ng/ml after 3 months post-transplantation). At the end of a 6-months follow-up of TAC therapy, associations of the *CYP3A5* SNP with drug concentrations (TAC-C_0_), daily dose normalized by weight (TAC-D mg/kg) and drug concentrations normalized by dose (TAC- C_0_/D) were analyzed. MPA levels were regularly monitored and associations of the *MRP2* and *UGT1A9* SNPs with drug concentrations (MPA-C_0_), daily dose normalized by body surface (MPA-D mg/m2) and drug concentrations normalized by dose (MPA-C_0_/D) were analyzed at one time point, once stable immunosuppression had been achieved. The analysis of the area under the curve in 12 h normalized by dose (AUC_0–12h_/D) was conducted on 98 and 44 patients with stable immunosuppression based on TAC or MPA therapy, respectively. Relative frequencies of ABO and Rh blood groups demonstrated that the study population was representative of the Chilean population (data not shown).

The study was conducted according to the declaration of Helsinki and was approved by the Ethics Committees of the Universidad de Chile and Hospital Luis Calvo Mackenna. Patients with evidence of graft dysfunction during the first year post-transplantation, with change of immunosuppressive medication to azathioprine or cyclosporine A within the first 6 months after transplantation, or parents that denied participation were excluded.

### DNA Extraction and SNP Genotyping

Nucleic acids were extracted from whole peripheral blood samples using QIAmp DNA Blood Mini Kit (QIAGEN) or MagNA Pure Compact Nucleic Acid Isolation Kit I (Roche) following the manufacturer’s instructions. *CYP3A5*, *MRP2*, and *UGT1A9* were selected as genes with variants of interest for this study, because they have been proposed as the major factors to explain the variability related to plasma drug levels (TAC and MPA) and have shown similar results in different populations. The presence of the *CYP3A5* c. A6986G polymorphism (rs776746) was determined using the PCR-RFLP technique with specific primers (forward 5′-CAT GAC TTA GTA GAC AGA TGA-3 ′and reverse 5′-GGT CCA AAC AGG GAA GAA ATA-3′) and the restriction enzyme *SspI*, according to previously described protocols (Rong et al., 2010). The assessment of the polymorphisms *UGT1A9* 2152C > T (rs17868320), *UGT1A9* -275T > A (rs6714486) and *MRP2* -24C > T (rs717620) was performed with the TaqMan™ Drug Metabolism Genotyping Assay (ThermoFisher). All SNPs were processed on LightCycler^®^ 480II and analyzed with the LightCycler^®^ 480II software (Roche). Each assay included controls confirmed previously by Sanger sequencing for the three genotypes (homozygous reference allele, heterozygous, homozygous variant allele) if available and negative controls with an equal volume of nuclease-free pure water.

### Therapeutic Drug Monitoring: TAC and MPA

To measure plasma drug concentration, four blood samples of 2 ml were taken in EDTA tubes, corresponding to C_0_ (trough level before dose), C_1_, C_2_, and C_4_ time points. Whole blood levels of TAC were measured using the Abbott Architect i1000 immunoassay (Abbott Laboratories). Plasma levels of MPA were measured using a liquid-liquid extraction and high-performance liquid chromatography equipment with a diode array detector (Agilent 1260, Agilent Technologies). The value of TAC AUC_0–12h_ was calculated in 98 patients using the abbreviated equation: AUC_0–12h_ = 10 + 1.4∗C_0_ + 0.8∗C_1_ + 1.6∗C_2_ + 5.5∗C_4_) (Wong et al., 2000). The value of MPA AUC_0–12h_ was calculated in 44 patients using the abbreviated equation: AUC_0–12h_ = 8.217 + 3.163∗C_0_ + 0.994∗C_1_ + 1.334∗C_2_ + 4.183∗C_4_ (Filler, 2004). The AUC_0–12h_ values were normalized by dose (TAC or MPA) for the following reasons: 1) AUC_0–12h_ was performed in each patient according to the clinical need to determine empirically drug exposure and was required at different time points, before and after 3 months of transplantation, that differ in AUC_0–12h_ and C_0_ therapeutic targets, requiring higher doses in the first 3 months period; 2) the study cohort is a pediatric population with different etiologies/co-morbidities and their ages ranged from 1.5 to 15.3 years at the time of transplantation, causing differences in drug metabolism that confer, in consequence, variability in dose requirements to reach therapeutic targets.

### Statistical Analysis

Pearson chi-square (χ^2^) goodness-of-fit test for the Hardy Weinberg equilibrium was applied to assess deviation of allele and genotype frequencies. Normality and homoscedasticity assumptions were checked for all variables or transformed with log to achieve normal distribution. AUC_0–12h_/TAC-D was analyzed by a one-way ANOVA to compare CYP3A5 genotypes. The analysis of AUC_0–12h_ without TAC dose normalization to compare *CYP3A5* genotypes are shown as supplemental material (Supplementary Figures S1, S2). TAC blood concentrations (TAC-C_0_), TAC daily dose requirement (TAC-D), TAC blood concentrations normalized by daily dose requirement (TAC- C_0_/D), and creatinine levels throughout the 6-months follow-up were analyzed by a repeated-measures ANOVA with genotype and time as main effects using the *ez* package (Lawrence, 2013). *Post hoc* tests were applied in the case of significant differences between groups by Tukey HSD test. MPA blood concentrations (MPA-C_0_), MPA dose requirement (MPA-D), and MPA blood concentrations normalized by dose requirement (MPA- C_0_/D) were analyzed by Wilcoxon-Mann Whitney test, grouping the heterozygous and homozygous carriers of the variant allele. The AUC_0–12h_/MPA-D values between carriers and non-carriers of the variant allele were compared with an unpaired one-tailed Wilcoxon-Mann Whitney test. The analysis of AUC_0–12h_ without MPA dose normalization to compare *UGT1A9 -275* variant carriers are shown as supplemental material (Supplementary Figure S3). Outlier values (mean ± 3*SD) suggestive of errors in sampling procedure, technical measurements or data manipulation were excluded from the analysis. Box plots show median and interquartile range (IQR) and whiskers represent the 1.5*IQR. All statistical analyses were performed with R (Wilson and Norden, 2015) and plots were made using *ggpubr* (Kassambara, 2020a) and *rstatix* (Kassambara, 2020b) packages for R. P-values < 0.05 were considered to be statistically significant.

## Results

### Characteristics of the Patients and Frequency of the *CYP3A5* Genotype

Altogether 104 pediatric kidney recipients from a single referral transplant center were included in this study. Clinical characteristics and *CYP3A5* genotype data are shown in Table 1. The median age at the time of transplantation was 8.1 years, and the leading cause of the end stage renal disease to require transplantation was a structural anomaly.

**TABLE 1:** Basic clinical characteristics and *CYP3A5* genotype of the 104 pediatric kidney recipients.

Characteristics	
Male/Female	52/52
Cause of ESRD	
Structural anomaly	70 (67.3%)
Glomerulopathy	14 (13.5%)
Monogenic cause (confirmed)	8 (7.7%)
Vascular cause	4 (3.8%)
Other	6 (5.8%)
Undetermined or unknown	2 (1.9%)
Age median at time of transplantation (years) [IQR]	8.1 [4.5–11.6]
Weight median at time of transplantation (kg) [IQR]	25.5 [15.9–35.2]
*CYP3A5* genotype	
CYP3A5 *1/*1	9 (8.7%)
CYP3A5 *3/*1	30 (28.8%)
CYP3A5 *3/*3	65 (62.5%)

The SNP rs776746 in *CYP3A5* was analyzed in the entire cohort and we found that 8.7% presented the CYP3A5*1/*1 genotype, 28.8% the heterozygous CYP3A5*1/*3 genotype and 62.5% the CYP3A5*3/*3 genotype (Table 1). The frequency of the CYP3A5*3 variant allele resulted in 76.9%. Genotype and allele frequencies were not significantly different than expected if the population was in Hardy-Weinberg equilibrium (χ^2^ = 3.66, *p* = 0.06). Compared to the Latino/Admixed American population reported in the gnomAd database v2.1.1 in December 2020 (https://gnomad.broadinstitute.org/), no statistical differences were found in our population in terms of genotype (χ^2^ = 3.09, *p* = 0.21) or allele (χ^2^ = 0.49, *p* = 0.48) frequencies of this SNP.

### Associations of the CYP3A5 Genotypes with AUC_0–12h_/D, Trough Levels (C_0_), Dose Requirements (D) and C_0_/D in TAC Therapy.

**FIGURE 1:**
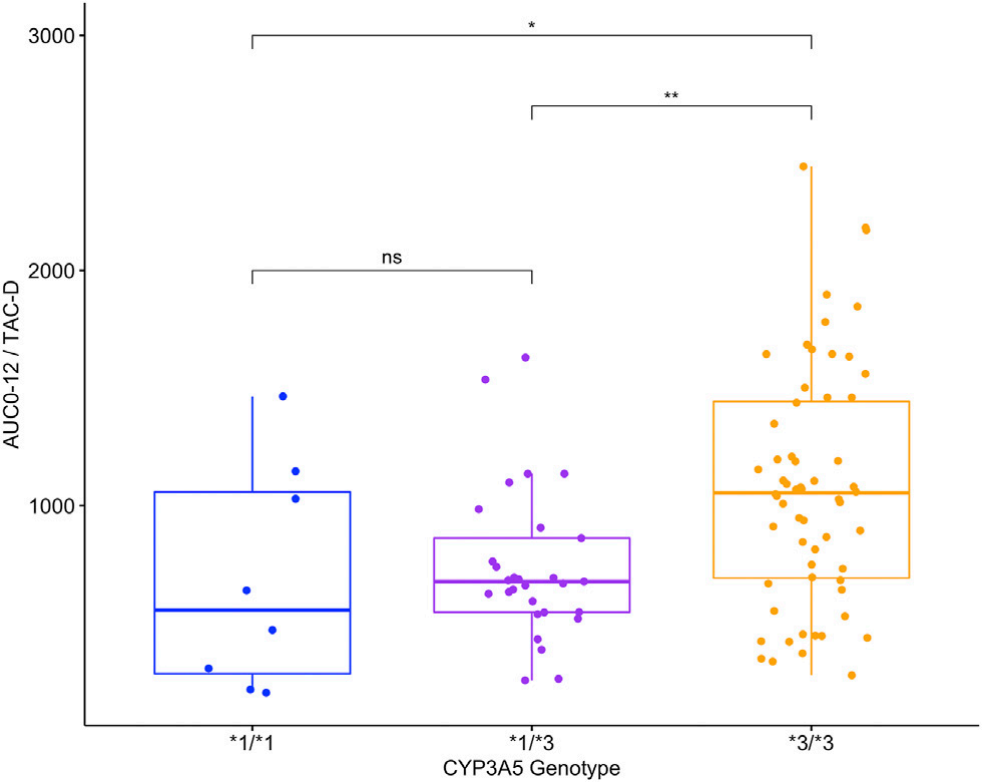
Area under the curve in 12 h normalized by TAC dose requirements (AUC_0-12h_/TAC-D ng*hr/ml/mg/kg) in 97 patients with different *CYP3A5* genotypes.

The analysis of AUC_0–12h_/TAC-D in 97 patients revealed significant differences (*p* < 0.01) among the *CYP3A5* genotypes (Figure 1). The CYP3A5*3/*3 patients showed the highest values (1058 [IQR 688.6–1460] ng*hr/ml/mg/kg), which was 1.56-fold higher than CYP3A5*1/*3 patients (676.9 [IQR 546.0–883.6] ng*hr/ml/mg/kg; *p* < 0.01) and 1.91-fold higher than CYP3A5*1/*1 patients (554.7 [IQR 239.7–1117]ng/h*ml/mg/kg; *p* < 0.05), respectively. AUC_0–12h_/TAC-D values did not differ between CYP3A5*1/*3 and CYP3A5*1/*1 patients (*p* = 0.79).

**FIGURE 2:**
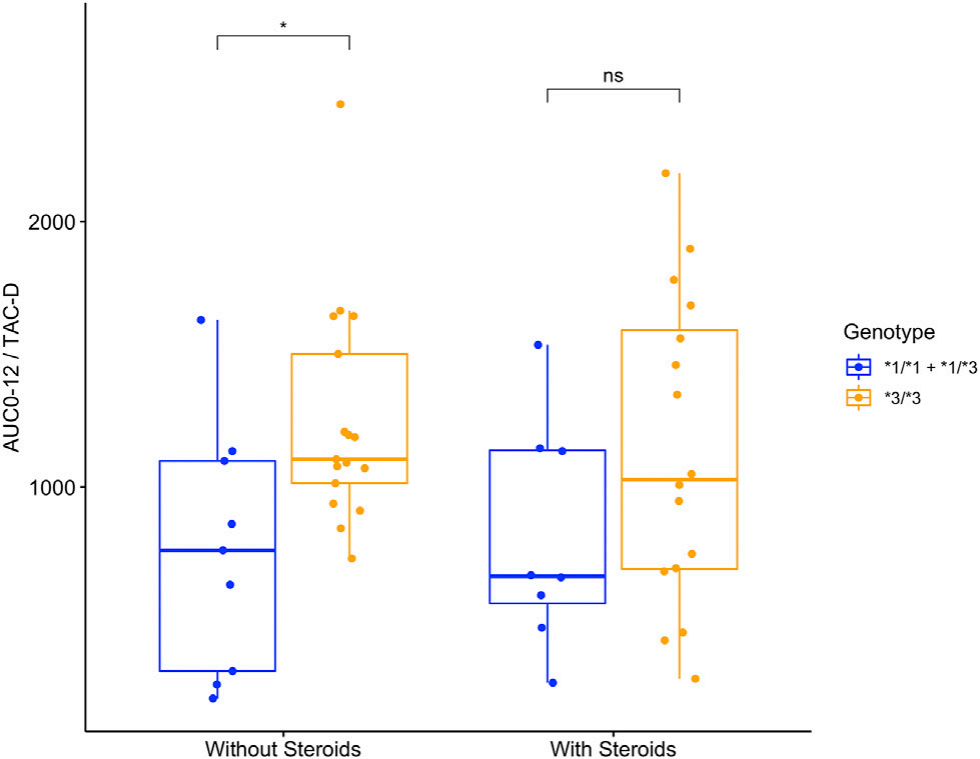
Area under the curve in 12 h normalized by dose requirements (AUC_0-12h_/TAC-D ng*hr/ml/mg/kg) in 52 patients with different *CYP3A5* genotypes with or without steroids from day 7 post-transplantation onwards.

Regarding the steroid therapy, the analysis showed some differences between *CYP3A5* genotypes (Figure 2). When analyzing patients with early steroid withdrawal, the carriers of the CYP3A5*3/*3 genotype showed AUC_0–12h_/TAC-D values (1099 [IQR 930.7–1537] ng*hr/ml/mg/kg), 1.58-fold significantly higher than the carriers of the CYP3A5*1 ancestral allele (CYP3A5*1/*3 n = 7 and CYP*1/*1; *n* = 2) (697 [IQR 404.5–1108] ng*hr/ml/mg/kg) (*p* < 0.05). No statistical differences were observed between *CYP3A5* genotypes that continued steroid therapy (*p* = 0.34).

**FIGURE 3:**
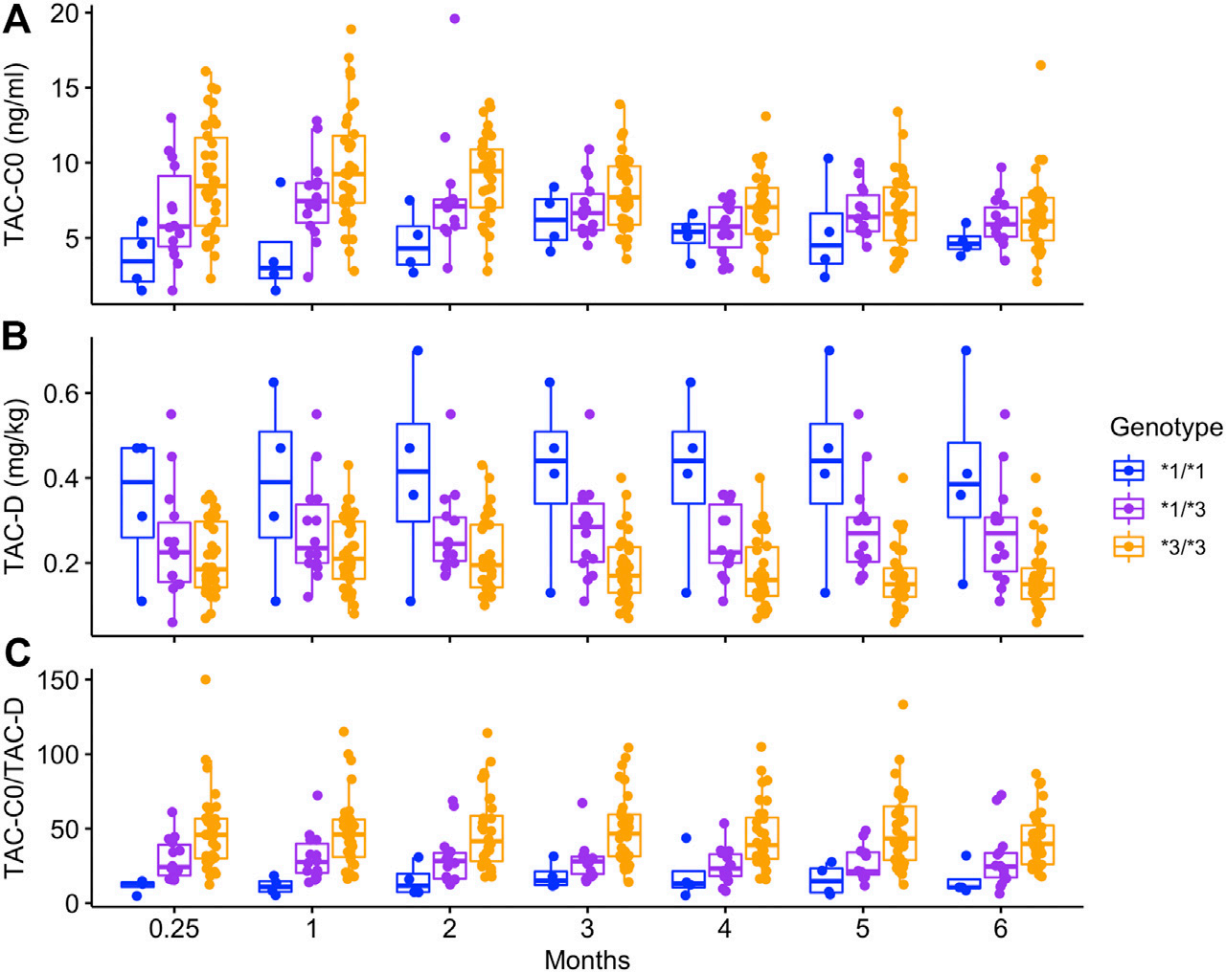
Trough levels (TAC-C_0_ ng/ml), dose requirements (TAC-D mg/kg) and trough levels normalized by dose requirements (TAC-C_0_/D ng*kg/ml/mg) in 52 patients with different *CYP3A5* genotypes in TAC therapy that underwent a 6-month follow-up.

In the analysis performed in 52 patients with 6 months of follow-up, the TAC blood concentrations (TAC-C_0_), TAC daily dose requirement (TAC-D) and TAC blood concentrations normalized by dose requirement (TAC- C_0_/D) differed significantly between the *CYP3A5* genotypes (TAC-C_0_ *p* < 0.001, TAC-D *p* < 0.01, TAC- C_0_/D *p* < 0.001) (Figures 3A–C). Specifically, patients with the CYP3A5*3/*3 genotype showed higher TAC-C_0_ values than patients with the CYP3A5*1/*3 and CYP3A5*1/*1 genotypes (*p* < 0.01 and *p* < 0.001, respectively). Additionally, patients with the CYP3A5*1/*3 genotype also exhibited higher TAC-C_0_ values than patients with the CYP3A5*1/*1 genotype (*p* < 0.001). Monitoring within the first three months, showed that 5–15% of the patients carrying the CYP3A5*1 allele had achieved target levels, lower than the 22–43% of the patients with the CYP3A5*3/*3 genotype. At the sixth month, 44.6% of the patients had achieved TAC-C_0_ in the range between 5 and 7 ng/mL, regardless of their *CYP3A5* genotype (Figure 3A). According to the analysis of TAC-D, patients with CYP3A5*1/*1 genotype showed higher TAC-D values than patients with the CYP3A5*1/*3 genotype and patients with the CYP3A5*3/*3 genotype (*p* < 0.01 and *p* < 0.001, respectively). Additionally, patients with the CYP3A5*1/*3 genotype also displayed higher TAC-D values than patients with the CYP3A5*3/*3 genotype (*p* < 0.001). Interestingly, significant differences between genotypes were not detected until the third month of follow-up, which is corroborated by an interaction between genotype and time (*p* < 0.01) (Figure 3B). Finally, patients with the CYP3A5*3/*3 genotype showed higher TAC-C_0_/D values than patients with the CYP3A5*1/*3 and CYP3A5*1/*1 genotypes (*p* < 0.001 in both cases). Similar to the observations in the TAC- C_0_ analysis, patients with the CYP3A5*1/*3 genotype also exhibited higher TAC-C_0_/D values than patients with the CYP3A5*1/*1 genotype (*p* < 0.001) (Figure 3C). No significant temporal variation (TAC-C_0_ *p* = 0.32; TAC-D *p* = 0.43; and TAC- C_0_/D *p* = 0.44) or genotype-time interaction were observed (TAC-C_0_ *p* = 0.18; and TAC- C_0_/D *p* = 0.56), except for the genotype-time interaction for TAC-D (see above).

The change in dose requirements comparing the initial prescribed TAC dose with the required TAC dose at the end of the 6-months follow-up was different between *CYP3A5* genotypes, experiencing a 209% increase in carriers of the CYP3A5*1 ancestral allele (CYP3A5*1/*3 and CYP*1/*1 patients; 0.314 ± 0.151 mg/kg, *p* < 0.01). By contrast, the TAC dose requirement of CYP3A5*3/*3 patients remained unaltered at the end of the follow-up in comparison to the initial prescribed TAC dose for these patients (0.164 ± 0.073 mg/kg, *p* = 0.99). Creatinine clearance was stable during the 6 months, regardless of the genotype (*p* = 0.76) (data not shown).

### Association of *MRP2* and *UGT1A9* Alleles with AUC_0–12h_/D, Trough Levels (C_0_), Dose Requirements (D) and C_0_/D in MPA Therapy.

When analyzing the SNPs in *MRP2* and *UGT1A9* that might influence MPA pharmacokinetics in a subgroup of 53 patients, genotype and allele frequencies were not significantly different than expected if the population was in Hardy-Weinberg equilibrium (*MRP2* -24C > T χ^2^ = 1.54, *p* = 0.21; *UGT1A9* -275T > A χ^2^ = 0.26, *p* = 0.61; *UGT1A9* -2152C > T χ^2^ = 0.046, *p* = 0.83), although a low frequency of some variant alleles was observed (Table 2). In particular, the frequencies of the *MRP2* -24A, *UGT1A9* -275A and *UGT1A9* -2152T alleles were 22.1, 6.6, and 2.9%, respectively. No significant differences between genotypes were observed in MPA-C_0_, MPA-D or MPA-C_0_/D (*MRP2* -24C > T: MPA-C_0_ *p* = 0.93, MPA-D *p* = 0.66, MPA-C_0_/D *p* = 0.86; *UGT1A9* -275T > A: MPA-C_0_ *p* = 0.27, MPA-D *p* = 0.50, MPA- C_0_/D *p* = 0.54; *UGT1A9* 2152C > T: MPA-C_0_ *p* = 0.30, MPA-D *p* = 0.99, MPA- C_0_/D *p* = 0.36).

**TABLE 2:** Basic clinical characteristics and *MRP2*/*UGT1A9* genotypes of 53 pediatric kidney recipients.

Characteristics	
Male/Female	28/25
Cause of ESRD	
Structural anomaly	35 (66.0%)
Glomerulopathy	5 (9.4%)
Monogenic cause (confirmed)	5 (9.4%)
Vascular cause	3 (5.7%)
Other	2 (3.8%)
Undetermined or unknown	3 (5.7%)
Age median at time of transplantation (years) [IQR]	8.8 [4.1–11.2]
Body surface median at time of transplantation (m2) [IQR]	1.01 [0.66–1.24]
*MRP2* -24G > A genotype^a^	
-24G/-24G	30 (57.7%)
-24G/-24A	21 (40.4%)
-24A/-24A	1 (1.9%)
*UGT1A9* -275T > A genotype	
-275T/-275T	46 (86.8%)
-275T/-275A	7 (13.2%)
-275A/-275A	0 (0%)
*UGT1A9 -2152C > T* genotype^a^	
-2152C/-2152C	49 (94.2%)
-2152C/-2152T	3 (5.8%)
-2152T/-2152T	0 (0%)

^a^ In one sample the genotype was not determined.

**FIGURE 4:**
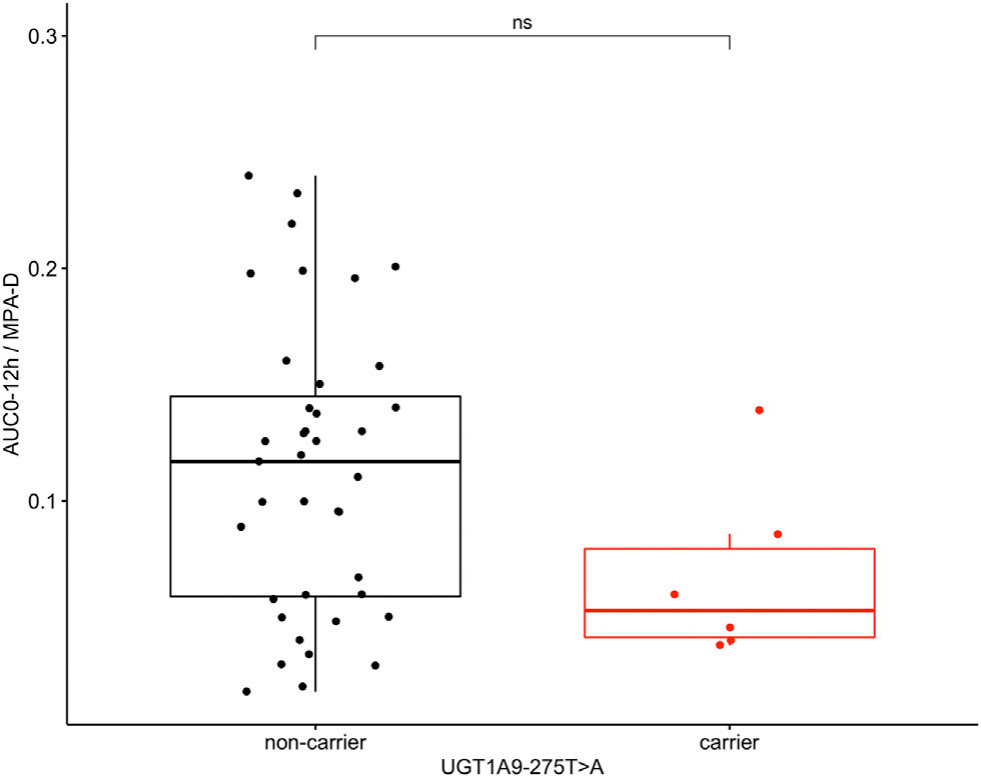
Area under the curve in 12 h normalized by MPA dose requirements (AUC_0-12h_/MPA-D ug*hr/ml/mg/m2) in 45 patients as non-carriers or carriers of the *UGT1A9*-275T>A variant allele.

Since the *UGT1A9* and *MRP2* alleles have been reported as variants associated with higher enzyme and transporter activities and higher MPA dose requirements, AUC_0–12h_/MPA-D was compared between carriers and non-carriers of the variant alleles. Interestingly, patients carrying the *UGT1A9*-275A variant allele had lower AUC_0–12h_/MPA-D (0.053 [IQR 0.040–0.100] ug*hr/ml/mg/m2) than patients carrying only the *UGT1A9*-275T ancestral allele genotype (0.117 [IQR 0.058–0.150]ug*hr/ml/mg/m2) with a difference of marginal significance (*p* = 0.05) (Figure 4). This difference was not observed in the other *UGT1A9* (*p* = 0.33) or *MRP2* (*p* = 0.29) alleles explored in this study.

## Discussion

An individualized immunosuppressive therapy is essential to minimize risks after transplantation. TAC is used as the first-line calcineurin inhibitor in many centers performing kidney transplantation in pediatric recipients given the evidence of its clinical effectiveness in preventing acute rejection and optimizing the function of the transplanted kidney (KDIGO Transplant Work, 2009). The main enzyme responsible for the TAC metabolism is *CYP3A5* and contains a well-studied genetic variant, CYP3A5*3, which represents the most frequent allele associated with a non-functional CYP3A5 enzyme worldwide (Kuehl et al., 2001).

According to our results, the CYP3A5*3 allele seems to be predominant in the Chilean population, which could be due in part to the genetic structure of Chileans as a consequence of the admixture between Native Amerindians, the historical migration of individuals from Europe and, to a lesser extent, the arrival of African individuals (Eyheramendy et al., 2015). Similar results have been observed in other admixed Latin American populations in terms of the presence of the three *CYP3A5* genotypes, the association with different TAC pharmacokinetic patterns and the influence of ancestry (Ferraris et al., 2011; Cusinato et al., 2014; Galaviz-Hernández et al., 2020). GWAS studies in North American, Caucasian, African and Asiatic populations have validated the role of *CYP3A5* as the main predictor of TAC metabolism, although a few other common variants in cytochrome P450 enzymes and drug transporters have been suggested as contributing to the variability in plasma drug levels (Oetting et al., 2018; Bruckmueller et al., 2015; Oetting et al., 2016). Latin American populations constitute a challenge with a hidden genetic complexity and are highly underrepresented in clinical-pharmacological research studies, but deserve to be analyzed to propose individualized therapies based on local experience for the pediatric as well as the adult population. The disparities in studies for some geographic and ethnic groups such as Latin American countries limit our understanding of their clinical phenotypes, and reduce access to personalized medicine to improve health care. In addition, association studies between polymorphisms and drug levels are more frequently performed on adult populations, with less evidence for pediatric populations as we present in this study.

In this study we decided to normalize AUC_0–12h_ by dose to reduce differences between patients and their characteristics at the time of transplantation, and the fact that AUC calculations were performed according to the clinical requirement to determine drug exposure that occurred at different days in each patient (before or after 3 months post-transplantation). The AUC_0–12h_/TAC-D values were higher for CYP3A5*3/*3 carriers and remained high in patients with steroid withdrawal. This is particularly important since the AUC calculation is considered an adequate marker for drug exposure and high values are associated with chronic nephrotoxicity. However, steroid withdrawal in patients using TAC and MPA as immunosuppressive therapy might be valuable to achieve better long-term graft function and patient survival by preventing hypertension, impaired glucose metabolism, growth retardation, obesity and infections, among other adverse effects (Sarwal et al., 2001; Gülhan et al., 2014). Although some patients presented high AUC_0–12h_/TAC-D values, we saw no evidence of supratherapeutic immunosuppression during the 6-months follow-up. On the other hand, the CYP3A5*1 allele was less predominant (23.1%), but strongly associated with lower AUC_0–12h_/TAC-D values without evidence of subtherapeutic immunosuppression during the follow-up and lower trough TAC levels. In fact, within the first three months fewer than 15% of patients carrying the CYP3A5*1 allele had reached target levels, suggesting that these patients require fine monitoring and that they may steroid therapy during the first post-transplantation period to prevent graft rejection.

TAC pharmacokinetics were strongly associated with the *CYP3A5* genotype in the study population. Of note, empirical dose adjustment during the follow-up showed substantial differences from the third month onwards between genotypes, with the daily dose requirement being 1.6–1.9 fold higher in CYP3A5 expressors (CYP3A5*3/*1 and CYP3A5*1/*1) compared to CYP3A5 non-expressors (CYP3A5*3/*3). This observation is consistent with the CPIC (Clinical Pharmacogenetics Implementation Consortium) guideline for TAC dosing, which recommends increasing the starting dose by 1.5–2 times in children and adolescents CYP3A5 expressors followed by therapeutic drug monitoring as recommended for adults (Birdwell et al., 2015).

The influence of genetic variants on MPA pharmacokinetics has been less validated than those related to TAC pharmacokinetics, which might be explained in part by the low frequency of variants in metabolizing enzymes and transporters. According to our data, the SNP in the *UGT1A9* promoter region, *UGT1A9* -275T > A, presents low allele frequency (6.6%) and was associated with lower MPA exposure due to higher glucouronidating activity in the liver as proposed previously in the literature (Girard et al., 2004). A larger cohort is needed to continue validation of *MRP2* and *UGT1A9* variants or explore non-genetic determinants to better understand MPA pharmacokinetics in pediatric kidney recipients (Hesselink and Van Gelder, 2005).

Our study had certain limitations, such as not having considered the haplotypes or the co-administration of TAC and MPA in the analysis. Given the limited number of participants and the low frequency of some alleles, the haplotype analysis was not included in this study, because the statistical analysis might not meet the targets. In addition, the combined use of TAC, MPA and steroids is recommended in transplantation of solid organs to prevent graft rejection, but several studies have suggested that drug interaction may occur when TAC and MPA are combined. In fact, a lower TAC clearance was observed in healthy volunteers when this drug was co-administered with MPA, indicating that enzymes and transporters are involved in the metabolism of both drugs (Kim et al., 2018). However, this drug-drug interaction is still controversial and a larger dataset is required to test this effect to prevent under- or over-immunosuppression in patients of different ethnic and geographic origin (Kagaya et al., 2008; Park et al., 2009).

According to the evidence, the expression of hepatic enzymes varies with age. During the fetal period, CYP3A7 predominates being replaced gradually after birth by CYP3A4 and CYP3A5. As a consequence, it may be that CYP3A5 protein has different effect on drug metabolism in pediatric patients at different ages. Although the analysis of ontogeny was not presented as part of this study, we compared patients according to the Tanner score (prepubescent vs pubescent patients) and found no significant differences between them that could be a consequence of the limited dataset of pubescent patients (data not shown). However, it might be hypothesized that variants in *CYP3A5* cause multiple clinical phenotypes, generating a wide spectrum that ranges from low expressor-poor metabolizer in younger patients to high expressor-rapid metabolizer phenotypes in older patients.

On the other hand, we are aware of the need to perform a long-term follow-up in association with genetic determinants to describe the presence of adverse events and/or acute graft rejection, since these situations might evolve from sub- or supra-therapeutic immunosuppression with no evident clinical manifestations in months. The use of biomarkers in the pre-transplantation and post-transplantation periods related to pharmacodynamic effects has been proposed as a complementary tool to predict the effects of immunosuppression (Wieland et al., 2010).

Finally, we could not rule out the nonadherence that is known to be prevalent among pediatric transplant recipients and that places them at risk of rejection and graft loss, especially those of older age and specific sociodemographic characteristics (Dew et al., 2009). Medication adherence can be measured by evaluating the fluctuation of plasma drugs levels with a median that results usually below the target levels (Fredericks et al., 2007). Nonadherence was not analyzed in this study population, but the *CYP3A5* and *UGT1A9* genotype assessment might give a better understanding of the patients’ metabolism and particularly in those of high enzymatic activity with low trough levels and high dose requirements to enhance credibility of their adherence to immunosuppressive drugs.

In conclusion, our results reinforce the need to consider the *CYP3A5* and *UGT1A9* genotype analysis in local transplantation guidelines for pediatric recipients that could be assessed while on the waiting list. Additionally, the routine parameter C_0_/D as part of therapeutic drug monitoring and drug adherence could be better interpreted according to the genotype to guide TAC and MPA dosing safely and perform a fine follow-up of patients at risk of adverse events (viral infections, malignancies, graft rejection). In the future, further analysis will be needed to evaluate the impact of early TAC and MPA dose adjustment according to the *CYP3A5* and *UGT1A9* genotypes.

## Data Availability

The raw data supporting the conclusions of this article will be made available by the authors, without undue reservation, to any qualified researcher. The dataset Krall et al. 2021 TAC-MPA for this study can be found in the FigShare with DOI: 10.6084/m9.figshare.13574555.
