# Supplementary figures and images for: CYP3A5 and UGT1A9 Polymorphisms Influence Immunosuppressive Therapy in Pediatric Kidney Transplant Recipients
Source: Front Pharmacol. 2021 Apr 22;12:653525. doi: 10.3389/fphar.2021.653525 (PMC8100460; doi:10.3389/fphar.2021.653525)

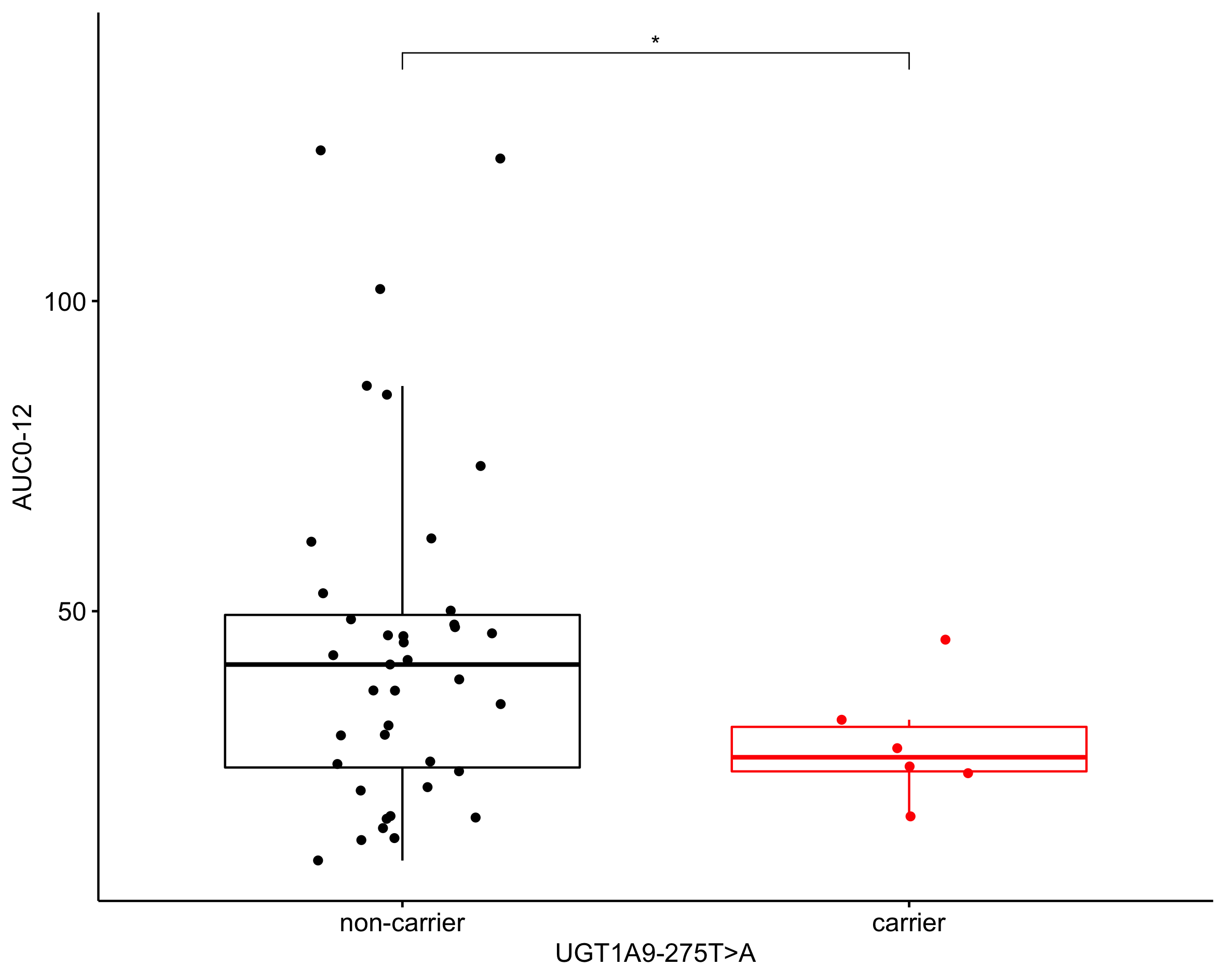

Supplement: Supplementary file 1 [file image3.tiff]

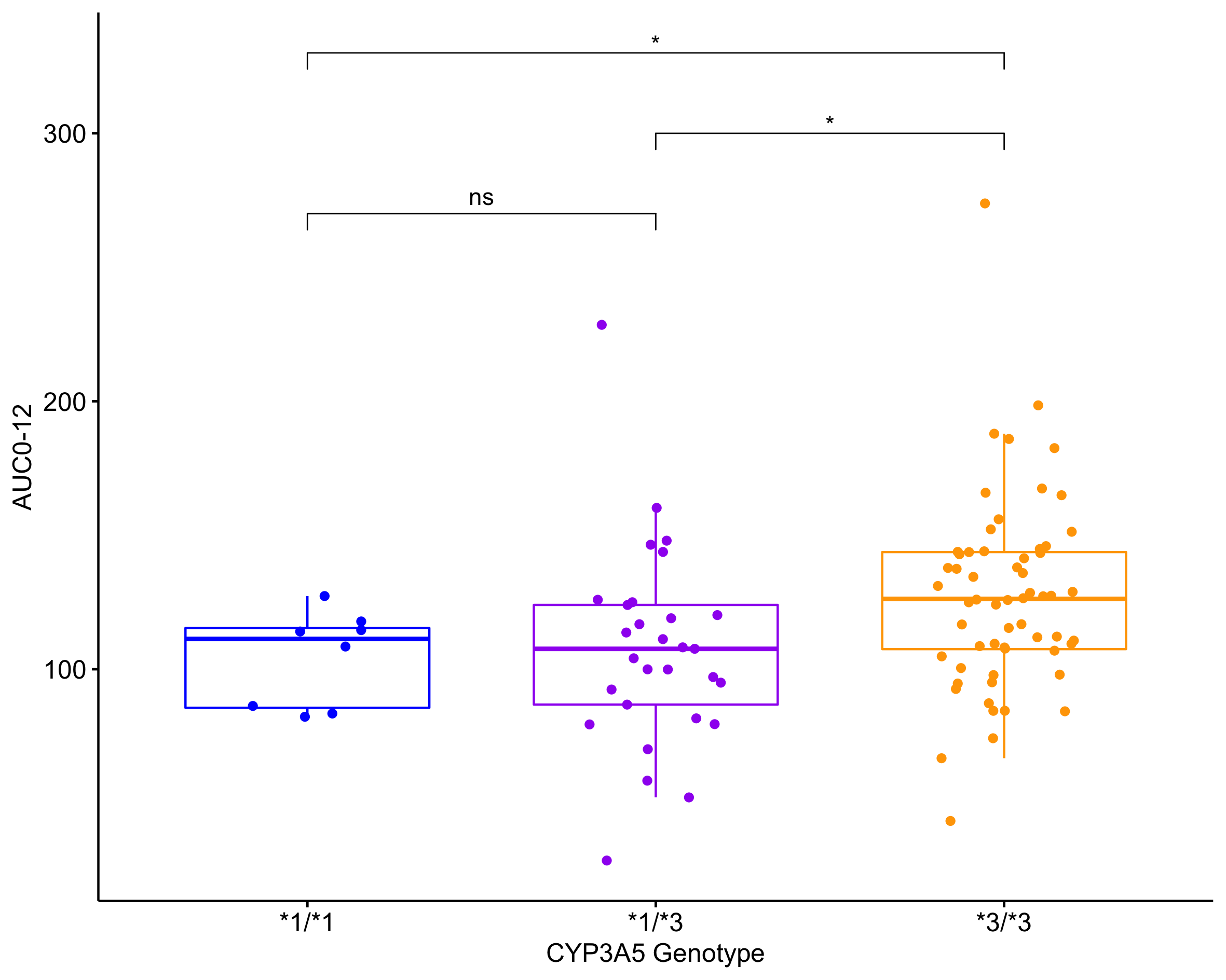

Supplement: Supplementary file 2 [file image1.tiff]

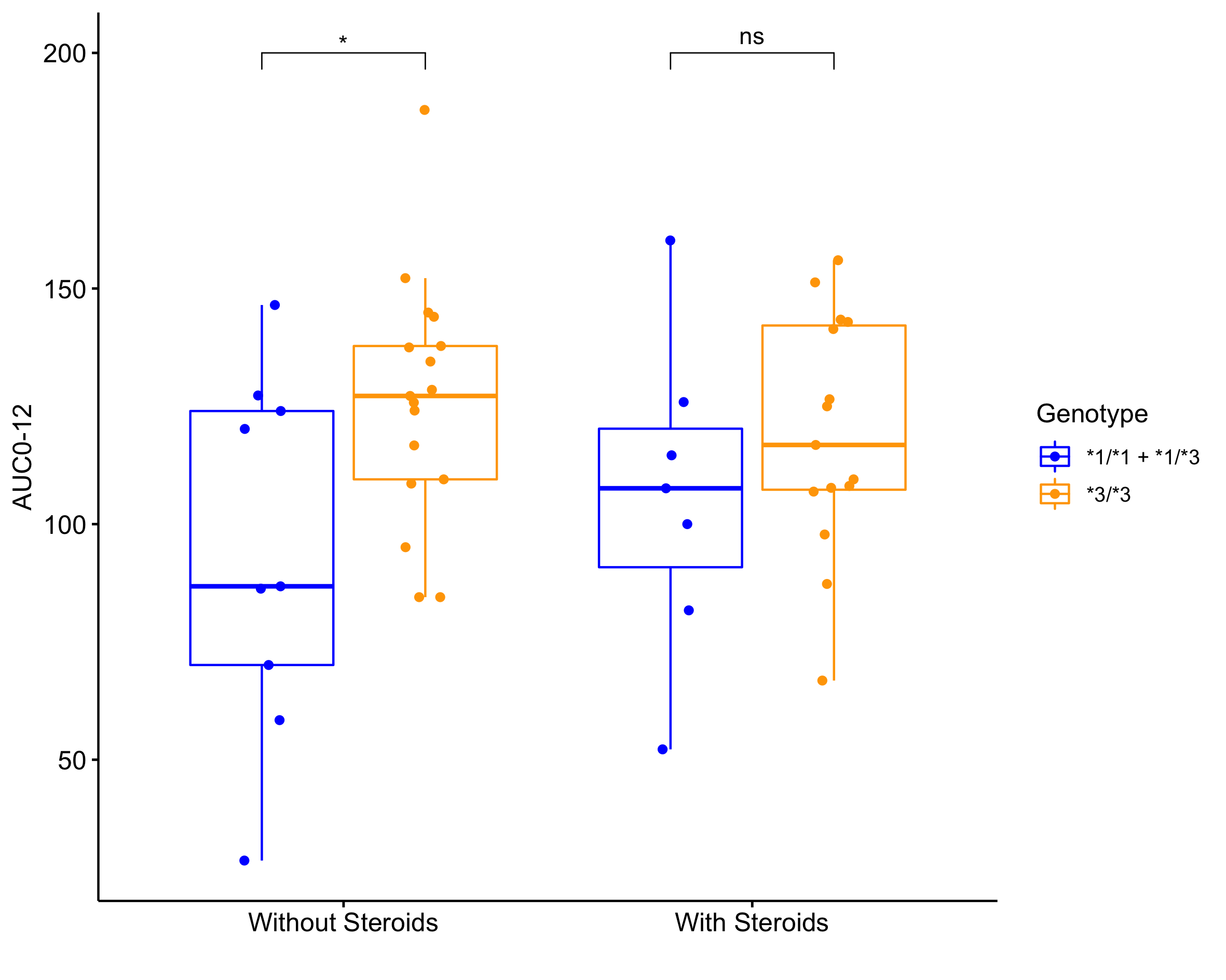

Supplement: Supplementary file 3 [file image2.tiff]
